# Crosstalk between glioblastoma and tumor microenvironment drives proneural–mesenchymal transition through ligand-receptor interactions

**DOI:** 10.1016/j.gendis.2023.05.025

**Published:** 2023-07-19

**Authors:** Yancheng Lai, Xiaole Lu, Yankai Liao, Pei Ouyang, Hai Wang, Xian Zhang, Guanglong Huang, Songtao Qi, Yaomin Li

**Affiliations:** a Department of Neurosurgery, Institute of Brain Disease, Nanfang Hospital, Southern Medical University, Guangzhou, Guangdong 510515, China; b Laboratory for Precision Neurosurgery, Nanfang Hospital, Southern Medical University, Guangzhou, Guangdong 510515, China

**Keywords:** Autocrine, Glioblastoma, Ligand-receptor interaction, Microenvironment, Paracrine, Proneural-mesenchymal transition

## Abstract

Glioblastoma (GBM) is the most common intrinsic and aggressive primary brain tumor in adults, with a median survival of approximately 15 months. GBM heterogeneity is considered responsible for the treatment resistance and unfavorable prognosis. Proneural-mesenchymal transition (PMT) represents GBM malignant progression and recurrence, which might be a breakthrough to understand GBM heterogeneity and overcome treatment resistance. PMT is a complicated process influenced by crosstalk between GBM and tumor microenvironment, depending on intricate ligand-receptor interactions. In this review, we summarize the autocrine and paracrine pathways in the GBM microenvironment and related ligand-receptor interactions inducing PMT. We also discuss the current therapies targeting the PMT-related autocrine and paracrine pathways. Together, this review offers a comprehensive understanding of the failure of GBM-targeted therapy and ideas for future tendencies of GBM treatment.

## Introduction

Glioblastoma (GBM) is one of the most common, intrinsic, and aggressive primary brain tumors in adults, which is characterized by diffuse infiltration throughout the brain, making it impossible to cure with surgery.^1^ The prognosis of recurring tumors has somewhat improved since the tumor treatment field (TTF) device in 2015 for the treatment of recurrent or refractory GBM.^2^ Immunotherapy, focused ultrasound, cell delivery methods, and nanomedicine are a few other recent medicinal advancements that have garnered interest.^3^^,^^4^ The quality of life and prognosis of GBM patients remain unfavorable, with an average median survival of approximately 15 months,^5^ despite advancements in treatment methods and supportive care. The difficulties that underlie therapeutic failure are primarily due to the heterogeneity of GBM. GBM heterogeneity is fueled by genetic, epigenetic, and microenvironmental factors that influence cellular processes.^6^ GBM has previously been divided into four subclasses by The Cancer Genome Atlas (TCGA) transcriptomics: proneural (PN), mesenchymal (MES), neural (NL), and classical (CL).^7^ With the establishment and improvement of single-cell RNA-sequencing (scRNA-seq), the NL subtype was identified as normal neural lineage contamination and dropped from the most recent GBM categorization.^8^

Proneural-mesenchymal transition (PMT), is the term used to describe the gradual change of GBM from the PN to MES subtype. PN is characterized by common mutations in IDH1, PDGFRA, and TP53, as well as a large amplification of chromosome 7 and deletion of chromosome 10 (>50%), which is associated with younger individuals.^8^ A more recent theory asserts that GBM cells can be divided into separate subregions, with PN subtypes distributed along the tumor margin.^9^ MES had significant expression of CHI3L1, MET, CD44, the MERTK, TNF-α superfamily, and NF-κB pathways, as well as inactivation of NF1 (37%), TP53 (32%), and PTEN (32%).^8^ This subtype is known to be associated with high angiogenesis and invasiveness, typically enriched in hypoxic, necrotic, and high glycolysis locations.^9^^,^^10^ STAT3, C/EBP, TAZ, NF-κB, Wnt, PI3K/AKT/mTOR, MAPK, and JNK in GBM cells are demonstrated to be directly associated with PMT.^11^, ^12^, ^13^, ^14^, ^15^, ^16^ Additionally, changes in the microenvironment (artificial treatment, metabolism, mRNA splicing) activate these pathways.^6^^,^^17^

GBM microenvironment is primarily made up of different types of solid tissue cells, soluble cytokines, and extracellular matrix (ECM).^18^ One of the primary mechanisms for cell communication in the microenvironment is receptor–ligand interaction. Receptors can be classified as membrane, cytoplasmic, or nuclear receptors based on the various binding locations.^19^ This review focuses on cell membrane receptors as important targets of neoadjuvant therapy, as they constitute a significant proportion. Tumor-associated macrophages (TAMs) are the predominant non-tumor cells in the environment.^18^ The anti-tumor M1 subtype and the pro-tumor M2 subtype are the two major subtypes in the GBM microenvironment.^20^^,^^21^ M2 TAM infiltration was identified as a significant contributor to PMT in the microenvironment.^21^

Moreover, angiogenesis, hypoxia, and high glucose are the keys to the variables that influence the conversion of TAMs to the M2 phenotype, which can lead to PMT and then harm patients.^22^^,^^23^ In addition, a number of other cells, including endothelial cells, T lymphocytes, oligodendrocytes, and mesenchymal stem cells, participate in the microenvironment's crosstalk through receptor–ligand interactions.^24^

The GBM microenvironment is complex, and understanding the relationships between cells and their interactions is crucial to understanding biological systems. Unfortunately, the current literature on ligand-receptor interactions driving PMT in the GBM microenvironment is fragmented. However, single-cell sequencing and spatial transcriptomics technologies have allowed for a shift in the study of cell–cell communication (CCC) towards understanding cell interactions rather than just the existence of cells. With the use of single-cell transcriptomics, new opportunities for exploring cell–cell communication have arisen.^25^ Recent studies have identified several mechanisms of tumor progression, such as chemotactic and cytokine signaling, context receptor signaling, survival and resistance signaling, DNA and membrane repair, avoidance of apoptosis induction, decreased drug efficacy, evasion of immunosurveillance, environmental control of survival, and cooperation of signaling programs.^26^ To better understand the development of tumors, it is important to consider the various types of tumors and the dynamic changes that occur in their internal and external environments.

Based on the new research demonstrated above, we frame the receptor-ligand interactions-based autocrine and paracrine pathways of the GBM microenvironment, offering a comprehensive understanding of the failure of GBM targeted therapy and ideas for future trends in GBM treatment.

## Autocrine pathways

Autocrine is when the same cell expresses the matching receptor protein on its surface as the ligand expressed by the sending cell. This mechanism is critical to the onset and development of various disorders.^25^ GBM cells secrete pro-oncogenic molecules that activate downstream signals through autocrine receptors, resulting in the maintenance of multiple malignant biological properties of GBM pathology.

The GBM-mediated autocrine pathways of PMT are complex. They involve autocrine ligands such as neurotransmitters, growth factors, chemokines, hormones, and other substances. Activation of multiple pathways, including NF-κB, JAK/STAT3, and PIK3/Akt, in tumor cells promotes PMT. These pathways control the mesenchymal transition and cause various biological functions, such as increased tumor cell invasion, altered metabolism, apoptosis inhibition, and angiogenesis, *etc*.

**Figure 1:**
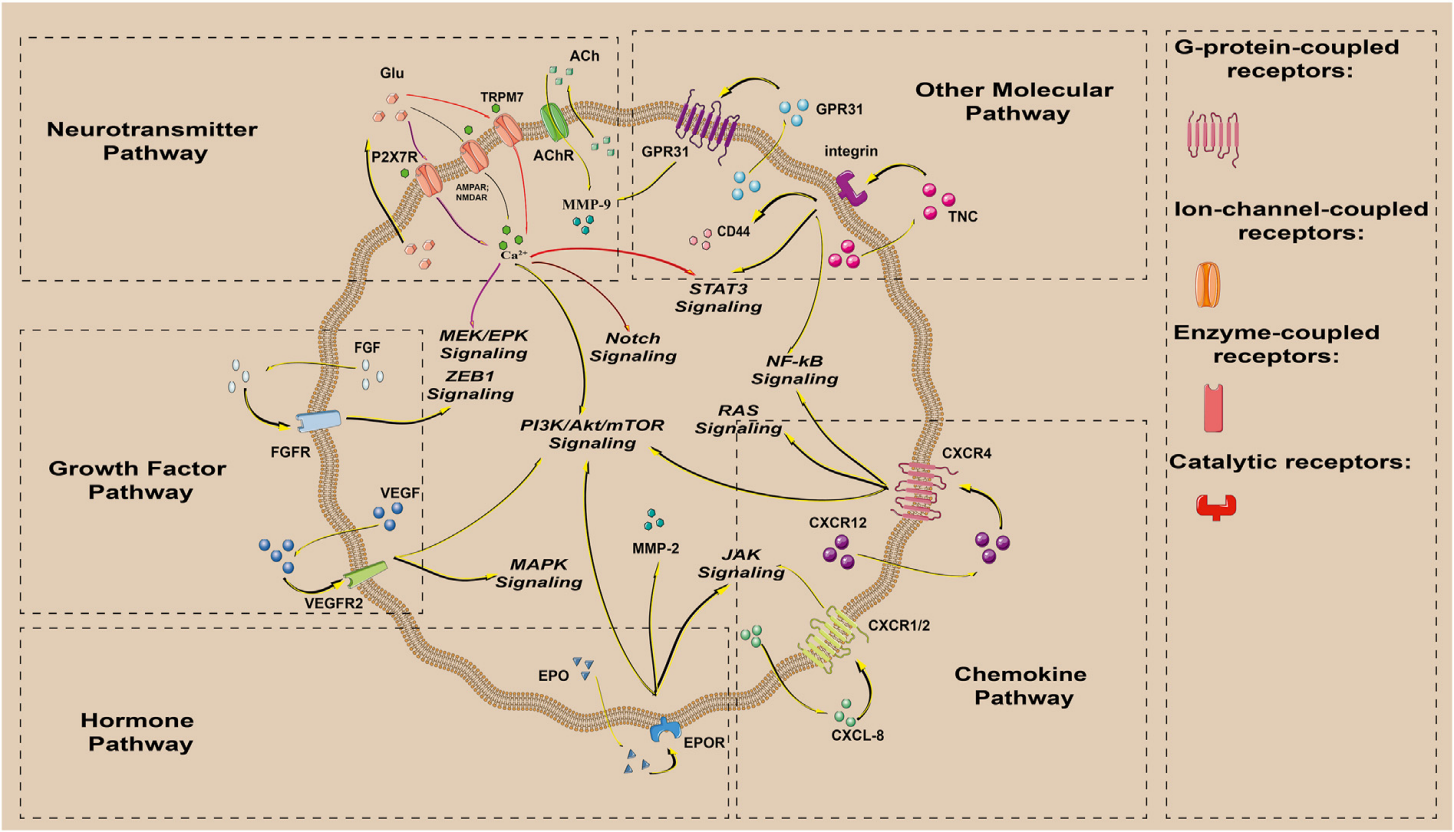
Autocrine mechanism controls PMT. Different ligands bind to their respective receptors to activate corresponding pathways and up-regulate the expression of related molecules to promote tumor progression in a mesenchymal manner.

The following is an introduction to some classic examples of recent research (Fig. 1 and Table 1).

**Table 1:** Molecular pathways of GBM autocrine promoting Proneural–Mesenchymal Transition.

Classification	Ligand	Receptor	Pathways activation	Molecular correlation	Hypoxia and necrosis
Neurotransmitter	Ach	AchR (M3)		MMP-9	
	Glu	NMDAR/AMPAR	Akt	Ca^2+^	
		TRPM7	STAT3/Notch		
		AMPAR	MEK/EPK		
	DA	DRD2/5	NF-κB	TF	+
	Ca2+	TRPC6		HIF-1α	+
Growth factor	VEGF	VEGFR2	MAPK/Akt	NRP1	+
	FGF	FGFR1-4	MAPK/PI3K/JAK	ADAMDEC1/ZEB1	
	PDGF	PDGFR	RAK/PI3K	TGF-β1	+
Chemokines	CXCL12	CXCR4	JAK/Akt/Ras	HIF-1α/VEGF/TNF-α/MMPs	+
	CXCL-8	CXCR1/2	JAK	CD44/Twist/HIF-1α	+
	OPN	IntegrinαVβ3	Akt/NF-κB	CD44	–
Hormone	EPO	EPOR	JAK/STAT/Akt	MMP-2	+
	AM	CLR/RAMP			+
	STC1	CASR	Akt/JNK	HIF-1α	+
Other Categories	TNC	Integrin	NF-κB/STAT3	CD44/TGF-β1/HIF-1α	+
	Ephrin-B2	EPHB4	Generate TDECs		
	12-HETE	GPR31	Akt	MMPs/ALOXE3	
	Chemerin	CMKLR1	NF-κB/STAT3	CD44/VIM	
	β2M	PIP5K1A	PI3K/Akt	TGF-β1	
	HMGB1	TLR/CXCR	Akt/EPK		+
	Rab27b	EGFR	NF-κB/STAT3/MAPK	EREG	–

### Neurotransmitter autocrine pathways

#### ACh and AChR (M3)

Neurotransmitter acetylcholine (ACh) is involved in synaptic transmission. The co-expression of choline transporter, choline acetyltransferase, and vesicular acetylcholine transporter in GBM cells was demonstrated by Thompson et al using information from the Repository for Molecular Brain Neoplasia Data (REMBRANDT) collection.^27^ The interaction between Ach and AChR (M3) was found to create an autocrine loop that promotes GBM invasion and triggers mesenchymal transition responses. Activation of AChR (M3) also enhances the activity of the matrix metalloproteinase MMP-9.^27^

#### Glu and ionic Glu receptor (NMDAR, AMPAR, TRPM7, P2X7R)

In the brain, glutamate (Glu) serves as a significant neurotransmitter.^28^ Glutamate (Glu) has been found to drive tumor PMT in GBM by activating calcium-permeable ion receptors, including AMPARs, NMDARs, TRPM7, and P2X7R, in an autocrine manner. Kyung-Seok Han et al have reported that Glu release from GBM cells increases intracellular Ca^2+^ levels.^29^ The activation of the Akt pathway is promoted by Ca^2+^ release mediated by AMPARs and NMDARs, while the activation of the Notch and STAT3 signaling pathways is promoted by the TRPM7 receptor, and the MEK/ERK pathway is promoted by the P2X7R receptor.^28^^,^^29^

#### Ca^2+^ and TRPC6

Transient receptor potential channel 6 (TRPC6) is a member of the TRPC family.^29^ By promoting a persistent rise in intracellular Ca^2+^ levels, which is essential for glioma proliferation and migration, induction of TRPC6 promotes the aggressive phenotype.^30^ Moreover, in hypoxic conditions, the hypoxia-inducible factor (HIF-1) is controlled by TRPC6 signaling, pointing to its connection with mesenchymal characteristics.^30^

#### DA and DRD2 or DRD5 (proneural subtype secretion)

The neurotransmitter dopamine (DA) is crucial for cerebral function.^31^ Dopamine receptor 2 (DRD2), one of the DA receptors, is overexpressed on the surface of GBM cells and exhibits a potent autocrine tumorigenic impact.^32^ Recent research has demonstrated that it is directly related to the development of GBM tumors. Targeted DRD2 therapies are also being developed.^33^ Additionally, according to data from Seamus et al, DA changed the metabolism of GBM cells to increase glycolysis, and the fact that DA is linked to hypoxia and the NF-κB pathway suggested a clear connection to mesenchymal properties.^34^

### Growth factor autocrine pathways

#### VEGF and VEGFR2 (KDR)

Angiogenesis is another characteristic of mesenchymal GBM. Vascular endothelial growth factor (VEGF), a well-known angiogenic growth factor, participates in PMT as a vital ligand.^35^ VEGFR2 (kinase insert domain receptor, KDR) mediates the autocrine pathway of VEGF in the GBM autocrine pathways.^35^ Knizetova et al proved that VEGFR2 is predominantly expressed on the cell surface of CD133^+^ human GSCs (glioma stem cells), whose viability, self-renewal, and tumorigenicity depend on signaling through the VEGF-VEGFR2-neuropilin-1 (NRP1) axis.^35^ As another significant pro-angiogenic factor, NRP1 binds to and stabilizes VEGFR2, enhancing the VEGF-VEGFR2 binding effect and promoting angiogenesis, furthering the pro-mesenchymal angiogenic action of VEGF-VEGFR2.^35^ Another study also has demonstrated that PMT-related pathways such as c-Raf/MAPK and PI3K/Akt are co-activated after ligand-receptor interaction of VEGF-VEGFR2.^36^

#### FGFs and FGFR1-4

It has been discovered that fibroblast growth factors (FGFs) control several processes, including growth, differentiation, survival, and migration.^37^ Four transmembrane receptor (FGFR1-4) tyrosine kinases work together to mediate the signaling caused by FGFs.^37^ The development, proliferation, and migration of GBM are all tightly correlated with the autocrine pathways of the FGF5/FGFR1-4 axis, which also appears to promote the expression of MAPK, PI3K, and JAK/STAT pathways.^37^

Additional research has demonstrated that FGF2/FGFR1 signaling is an autocrine positive feedback mechanism that supports angiogenesis, cell proliferation, and self-renewal in GBM.^38^ A disintegrin known as ADAMDEC1 (a disintegrin and metalloprotease domain-like decysin 1) has been demonstrated to positively regulate this autocrine loop.^38^ The research also demonstrated a favorable relationship between FGF and ZEB1. In the intracellular molecular pathway of PMT, ZEB1 is one of the important actors,^38^ which strongly suggests that FGF can encourage PMT.

#### PDGF and PDGFR

Another growth factor, platelet-derived growth factor (PDGF), mediating autocrine signaling through PDGF receptor (PDGFR) tyrosine kinases, regulates mitogenic pathways in GBM cells.^39^ Moreover, this autocrine loop has a high correlation with hypoxia, and VEGF receptors, which may indicate a connection with the production of mesenchymal-like characteristics.^40^ Furthermore, according to research by Stommel et al, PDGFR and EGFR (epidermal growth factor receptor) can co-express in GBM cells and activate RTK and PI3K signaling pathways, which promote the development and proliferation of tumors.^41^

### Chemokine autocrine pathways

#### CXCL8 and CXCR1/2

Interleukin-8 (IL-8), also known as CXCL8, is an important inflammatory mediator and chemokine that is functional through engaging with its receptors, CXCR1 and CXCR2.^42^ CXCL8 up-regulation has been seen in GBM, and it has been shown that Bcl-xl-induced CXCL8 up-regulation in GBM cells is mediated through the NF-κB-dependent mechanism, consistent with other tumor research.^43^ This NF-κB-dependent mechanism occurs as a result of the JAK/STAT1/HIF-1/Snail signaling pathways in tumor cells.^44^ Additionally, CXCL8 is related to several MES-related markers, including CD44 and Twist.^45^ Moreover, some studies have shown that necrotic cells in the tissue cause GBM cells to secrete CXCL8, induced by HIF-1-mediating hypoxia,^46^ which also indicates that CXCL8 is connected to mesenchymal characteristics.

#### CXCL12 and CXCR4

Chemokine ligand 12 (CXCL12) is one kind of chemokine. It was shown by Gatti et al that GBM released CXCL12, which binds to the CXCR4 auto-receptor. Then, several biochemical pathways connected to PMT are activated and altered as a result of a change in the three-dimensional conformation of CXCR4. When the ligand was bound to the CXCR4 receptor, the receptor separated into the αi and βγ subunits.^47^ The downstream PMT master regulators including PYK2, NF-κB, JAK/STAT, and PIK3/Akt pathways were activated by the αi subunit. Similarly, βγ subunit modifies the cell cycle via activating the Ras pathway.^47^ Moreover, it has been demonstrated that the CXCL12/CXCR4 axis supports MMPs activity in GBM, suggesting a connection between these pathways and mesenchymal transition.^48^

The expression of CXCR4 and CXCR7 are the hallmark of mesenchymal GBM cells, which are typically found in the hypoxic necrotic regions of the tumor.^47^ The expression of CXCL12 and CXCR4 was also found to be boosted by HIF-1 to aid this autocrine loop.^47^ Moreover, numerous molecules, including VEGF, are expressed in tandem with the CXCL12/CXCR4 axis, which is regulated by TNF-α, IL-4, and IL-6.^49^

#### OPN and integrin αVβ3 or variant forms of CD44

Osteopontin (OPN) (also known as secreted phosphorylated protein-1, SPP1) is a chemotactic factor and a glycophosphoprotein.^50^ OPN is a ligand widely expressed by a variety of cell types, such as osteoblasts, macrophages, epithelial cells, smooth muscle cells, and cancer cells.^51^ The receptors of OPN include several CD44 isoforms as well as the integrins αVβ3, αVβ5, αvβ1, and αVβ1.^52^, ^53^, ^54^ The degree of angiogenesis and GBM is correlated with OPN expression levels.^55^

The link between OPN and mesenchymal transition is revealed via OPN-mediated autocrine and paracrine pathways. The continued stemness of tumor cells, which is sustained by the OPN/CD44 autocrine loop, triggers the Akt pathways.^56^ Additionally, there is proof that OPN also triggers the NF-κB pathway and that integrin receptors, including αVβ3, are responsible for this effect.^54^

### Hormone autocrine pathways

#### EPO and EPOR

Erythropoietin (EPO) is a hormone that increases red blood cell production.^57^ In GBM, EPO functions as PMT in an autocrine manner, which activates the JAK2, STAT5, and Akt pathways.^57^ Mohyeldin et al demonstrated that the hypoxic areas and invasive margins of glioma specimens acquired through biopsy showed expression of both EPO and EPOR, and the expression of EPOR was correlated with the tumor's stage.^58^ EPO supports angiogenesis, ensures the survival of cancer cells, and encourages their growth.^59^ EPO was distinguished by a high level of expression in the necrotic regions of GBM, facilitated tumor invasion via MMP-2, and regulated by HIF-α.^60^

#### AM and CLR/RAMP2, CLR/RAMP3

The adrenal glands produce the stress hormone adrenomedullin (AM).^61^ AM is linked to a subgroup of GBM that is expressed in regions of hypoxic necrosis.^62^ Additionally, studies have shown that AM promoted angiogenesis by interacting with its receptors, CLR/RAMP2 and CLR/RAMP3.^62^ Moreover, AM expression is positively associated with VEGF expression.^62^ These AM characteristics indicate its role in the mesenchymal transition.

#### STC1 and CaSR

Stanniocalcin-1(STC1) is a secreted glycoprotein hormone that, through autocrine and paracrine activities, mediates the PMT of GBM.^63^ The role of STC1 as a new promoter of GBM metastasis was found to be regulated by four microRNAs, including miR-29B, miR-34a, miR-101, and miR-137, as described by Sakata et al.^63^ Other research has demonstrated that STC1 activates cyclin 1 and cyclin-dependent kinase 2 (CDK2) to support proliferation in the autocrine loop.^64^ STC1 participated in the spread of GBM tumors by activating the PI3K/Akt and JNK signaling pathways.^65^ More significantly, HIF-1 promotes STC1 production in hypoxic environments.^64^

### Other molecular autocrine pathways

#### TNC and integrin or CD44

Tenascin C (TNC), an extracellular matrix that rarely expresses in adults and typically expresses during embryonic development.^66^ TNC is highly expressed in tumor cells and acts in a variety of ways by binding to integrin receptors.^66^ In the MES-GBM subtype with strong NF-κB signaling activity, TNC is found to be up-regulated, according to research by Angel et al.^67^ Furthermore, TNC was shown to be positively correlated with the expression of MES markers such as STAT3, TGF-β, and CD44, while negatively correlated with PN phenotypic markers like OLIG2, DLL3, and ASCL1.^67^ TNC was demonstrated to have a strong relationship with HIF-1, harming endothelial cells and encouraging GBM angiogenesis, invasion, and proliferation.^68^ TNC silencing attenuated GSCs' proliferation, migration, and renewal ability.^67^

The interplay of CD44 receptors in GBM and TNC in ECM further supports the link between PMT and TNC.^67^ Gupta et al showed that CD44 and TNC are co-expressed and linked to YBX1, a potential regulator of tumor invasion, in GBM.^69^ These demonstrate that GBM can be affected by TNC in the ECM to encourage mesenchymal transformation.

#### Ephrin-B2 and EPHB4

The TNC stated above can stimulate the expression of Ephrin-B2.^67^ Angel et al also discovered that TNC played a novel autocrine function in the plasticity of glioma cells and the production of TDECs (tumor-derived endothelial cells) by activating the NOTCH signaling pathway and up-regulating and secreting the pro-angiogenic EphrinB2 signaling axis.^67^ Other studies also verified that Ephrin-B2 is the main element of TNC-induced angiogenesis.^68^ Thus, TNC and Ephrin-B2 are significant GBM mesenchymal transition factors.

#### Chemerin and CMKLR1

Chemerin, also known as retinoic acid receptor responder protein 2 (RARRES2), is a released protein that interacts with CMKLR1 and influences the development of different tumors.^70^ The mesenchymal phenotype of GBM cells is reinforced by an autocrine and paracrine pathway that is mediated by chemerin, according to research by Wu et al.^71^ Chemerin, which is primarily expressed in TAMs and partially expressed in GBM cells, promoted the mesenchymal characteristics of GBM by inhibiting the ubiquitin-proteasomal degradation of CMKLR1, increasing NF-κB pathway activation in the process.^71^ Studies have demonstrated a strong positive correlation between the expression of mesenchymal markers such as N–Ca, CD44, and VIM and the expression of chemerin produced by GBM tumor cells.^71^ Additionally, the effect of chemerin on the mesenchymal transition can be increased by TNF-α production.^71^

#### β2M and PIP5K1A

A type of non-glycosylated protein called β2-microglobulin (β2M) plays a crucial role in the major histocompatibility complex-1 (MHC-1) for the presentation of antigens.^72^ Li et al showed that β2M was also a crucial regulator of GSC proliferation, maintenance, and self-renewal, through both autocrine and paracrine effects interacting with PIP5K1A and activating the PI3K/AKT/mTOR pathways.^73^ Additionally, β2M also promoted the synthesis and secretion of TGF-β1, demonstrating that it is a regulator essential for the mesenchymal transition.^73^

#### WISP1 and integrin α6β1

Wnt/β-catenin signaling is extremely active in GSCs in GBM, promoting tumor growth and malignant progression.^74^ Tao et al found that the Wnt-induced signaling protein 1 (WISP1) released by GSCs in GBM stimulated the remodeling of the tumor microenvironment and preserved the stem cell characteristics of GBM through autocrine and paracrine processes.^74^ WISP1 binds to the integrin α6β1 receptor in the autocrine pathways and stimulates the PIK3-Akt pathways to mediate self-renewal and proliferation, related to mesenchymal features.^74^ Another study discovered that inhibiting WISP1 induced apoptosis and cell cycle arrest in glioblastoma cells and inhibited their ability to proliferate, migrate, and invade.^75^ TAMs M2 polarization and persistence have also been shown to be supported by WISP1/α6β1 pathway (described below).

#### HMGB1 and TLR2/TLR4/RAGE/CXCR4/CXCR12

High mobility group box-1 protein 1 (HMGB1) is a DNA chaperone, which is often secreted by immune cells.^76^ HMGB1 has been linked to a number of biological activities, including DNA repair, transcription, cell proliferation, and migration.^76^ According to Cheng et al, HMGB1 is an independent prognostic biomarker for GBM patients.^16^ In the tumor microenvironment, HMGB1 released from GBM cells interacts with several receptors, including TLR2/TLR4 and RAGE.^16^ As a result of HMGB1-RAGE binding, the AKT and ERK signaling cascades were activated, which promoted GBM cell invasion.^16^ Another research demonstrated that the interaction of HMGB1 and TLR2/4 promoted NF-κB activation.^77^ Moreover, hypoxia also up-regulated the expression of HMGB1.^78^

#### EREG and EGFR

The oncogene Rab27b, a member of the Rab family, facilitates the growth and invasion of some kinds of tumors.^79^ Rab27b mediated the radioresistance of GBM cells, as demonstrated by Nishioka et al.^80^ Following radiation, Rab27b causes the release of epithelial regulatory protein (EREG), which interacts with EGFR to encourage the development of neighboring cells.^80^ The study also showed that PMT signaling pathways, such as NF-κB, STAT3, and MAPK, are downstream of EREG.^80^ Additionally, they demonstrated that the release of substances via the EREG/EGFR pathway following radiation modified the subtypes and/or transdifferentiation of cancer cells in the GBM tumor microenvironment,^80^ suggesting a connection to PMT. Other research has also demonstrated that the EREG/EGFR pathway, which is mediated by Rab27b, has significant impacts on cancer cell proliferation, survival, invasion, and microenvironment modification.^81^

## Paracrine pathways

**Figure 2:**
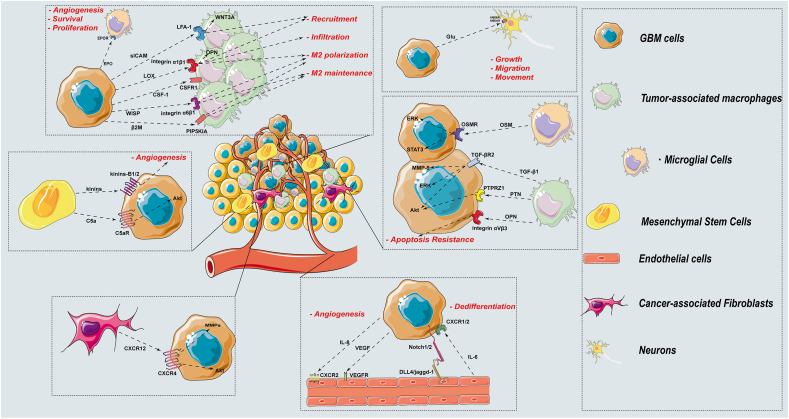
Paracrine mechanism controls PMT. This diagram demonstrates how cell–cell ligand-receptor binding affects PMT modulation in the GBM microenvironment. TAMs, endothelial cells, mesenchymal stem cells, and fibroblasts are the main paracrine mechanisms that engage with tumor cells in the GBM microenvironment.

Paracrine occurs when the transmitting ligand is expressed in a different cell than the matching recipient. GBM cells modify the microenvironment by secreting various molecules, including cytokines, chemokines, colony-stimulating factors, and growth factors. Meanwhile, normal cells in the microenvironment introduce pro-oncogenic molecules to paracrine receptors in GBM cells. This crosstalk through paracrine pathways constructs the foundation of GBM pathology by inducing angiogenesis, proliferation, invasion, and metastasis.^82^ This study summarizes the current paracrine pathways related to PMT in GBM and aims to provide direction for future research (Fig. 2 and Table 2).

**Table 2:** Molecular pathways of GBM paracrine promoting Proneural–Mesenchymal Transition.

Releaser	Ligand	Recipient	Receptor	Biological Function
GBM	WISP1	TAMs	integrinα6β1	M2 polarization and maintenance
	β2M		PIP5K1A	
	CSF-1		CSF-1R	
	Chemerin		CMKLR1	
	Ligand of MARCO		MARCO	
GBM	LOX	TAMs	integrinα1β1	Recruitment and infiltration
	sICAM		LFA-1	
GBM	OPN	TAMs	integrinαVβ3	Recruitment and M2 polarization
TAMs	TGF-β1	GBM	TGF-βR2	MMPs/Akt/EPK
	PTN	GBM	PTPRZ1	Akt
	OPN	GBM	integrinαVβ3	M2 polarization/Angiogenesis
TAMs (Microglia)	OSM	GBM/Oligodendrocytes	OSMR/LIFR	JAK/STAT3/ERK
GBM	CXCL8	Endothelial cells	CXCR2	Angiogenesis
	VEGF		KDR	
	CXCL12		CXCR4/7	
	FGF5		FGFR1	
	STC1		CaSR	
	Ephrin-B2		EPHB2	
Endothelial cells	DLL4	GBM	Notch1/2	Perivascular niche formation
	Jagge-1		Notch1/2	
	eNOs		Notch1/2	
	IL-6		CXCR1/2	
PN-GBM	TF	MES-GBM	TFR1	Iron death decline
GBM	EPO	Microglia/Oligodendrocytes	EPOR	Angiogenesis
GBM	Glu	Neuron	NMDAR/AMPAR	Proliferation and migration
MSCs	Kinins	GBM	Kinins-B1/B2	Angiogenesis/Migration
	C5a	GBM	C5aR1	ZEB1/MAPK
Fibroblasts (CAFs)	IL-6/11	GBM	IL6R/11R	JAK/STAT3
Fibroblasts	CXCL12	GBM/Endothelial cells	CXCR4	Angiogenesis/JAK/Akt
	HA	GBM	CD44/RHAMM	CD44
	TNC	GBM	CD44	CD44/TGF-β1/HIF-1α

### Paracrine pathways of GBM and tumor-associated macrophages

Tumor-associated macrophages (TAMs) are main non-tumor cells in the tumor microenvironment (TME), which have a complex paracrine pathway with GBM cells.^83^ In addition, it is confirmed that the mesenchymal phenotype and the M2 phenotype of TAMs are related significantly.^21^ Not only do GBMs through paracrine pathways recruit and polarize M2 TAMs, but M2 TAMs also through paracrine pathways facilitate the malignant mesenchymal progression in GBM.^22^^,^^23^ In conclusion, investigating the interactions between GBM and TAM might help to clarify PMT and provide fresh guidance for the treatment of GBM.

### Paracrine pathways of GBM recruiting and polarizing TAMs

#### sICAM and LFA-1 of TAMs

Soluble intercellular adhesion molecule-1 (sICAM-1) is a transmembrane glycoprotein that functions as an intercellular adhesion ligand.^84^ Yoo et al demonstrated that GBM cells generated sICAM-1 following radiation therapy, which worked in conjunction with the LFA-1 receptor on TAMs to encourage macrophage infiltration and enrich the tumor microenvironment with inflammatory macrophages.^84^ Additionally, their research demonstrated that the binding of sICAM and LFA-1 initiated the signaling pathway of WNT3A, a member of the family of airfoil-free MMTV integration sites, resulting in GBM mesenchymal transformation.^84^

#### LOX and integrin α1β1 of TAMs

Lysyl oxidases (LOX) secreted by GBM cells function as a potent macrophage chemoattractant via activation of the integrin α1β1-PYK2 pathway in macrophages.^85^ These infiltrating macrophages secrete OPN, which sustains glioma cell survival and stimulates angiogenesis (described below). In different research, Chen et al used functional studies and analysis of glioma cells in a GBM pleomorphism model to discover that PTEN deficiency activates YAP1, directly promotes the LOX/integrin α1β1 pathway, and concurrently increases the expression of GBM mesenchymal molecules CD44 and VIM.^85^

#### CSF-1 and CSF-1R of TAMs

The colony-stimulating factor-1 (CSF-1) released by GBM influences immunological aggregation and infiltration features by binding with the CSF-1R receptor of TAMs.^86^ Research has demonstrated that the combination of CSF-1 and CSF-1R can promote various pro-tumor anti-inflammatory responses, including maintaining the M2 phenotype of TAMs and accelerating the formation of GBM.^86^ These evidences point to a connection with GBM's mesenchymal characteristics.

#### WISP1 and integrin α6β1 of TAMs

PIK3-Akt is activated by the autocrine pathways of WISP1 to encourage GBM self-renewal and proliferation, as previously mentioned.^74^ Additionally, WISP1, which is secreted by GBM cells, interacts with the integrin α6β1 receptor on TAMs to support polarization and maintenance of M2-type TAMs.^74^ This offers more proof that WISP1 and PMT are related.

#### β2M and PIP5K1A, LILRB1 of TAMs

As demonstrated by Li et al, GBM cells produced β2M, interacting with PIP5K1A receptors on TAMs and activating the SMAD and PI3K/Akt signaling pathways.^73^ Crucially, this paracrine β2M/PIP5K1A pathway polarized TAMs to the M2 phenotype, mediating immune infiltration with mesenchymal characteristics.^73^ Furthermore, leukocyte immunoglobulin-like receptor B1 (LILRB1) on TAMs and β2M have been found to interact to send immunological escape signals.^73^

#### Chemerin and CMKLR1 of TAMs

Chemerin mentioned above also participates in the PMT-related paracrine pathway. Chemerin has been demonstrated to be involved in microenvironmental TAM recruitment by Wu et al.^71^ Moreover, it mediates the immunosuppressive milieu and encourages the conversion of the TAM phenotype to the M2 phenotype primarily through the CMKLR1/NF-κB axis.^71^ Furthermore, when TAMs are recruited and polarized by GBM overexpressing chemerin, inflammatory factors such as IL-1β and TNF-α, as well as immunosuppressive factors such as PD-L1 and TGF-β, are up-regulated.^71^^,^^87^ In conclusion, the chemerin/CMKLR1 axis plays a role in both the associated intracellular PMT pathways and the activation of the external immunosuppressive milieu.

#### OPN and integrin αVβ3 of TAMs or MDSCs

Through the paracrine pathway, GBM-released OPNs attach to integrin αVβ3 of TAMs to encourage the recruitment of macrophages and the differentiation of the mesenchymal phenotype through angiogenesis.^53^^,^^88^ As a result, TAMs produce more OPN and draw more TAMs into the microenvironment.^53^ Some studies claim that OPN can cause M2 polarization and sustain TAMs, but its primary purpose is to uphold and consolidate the M2 phenotype.^53^ Another discovery showed that by activating STAT3, the master factor of mesenchymal transition, OPN encourages the growth of myeloid-derived suppressor cells (MDSCs) and suppresses anti-tumor immunity.^89^

#### MARCO of TAMs

According to a recent study, recombinant macrophage receptor with collagenous structure (MARCO) in TAMs plays a role in PMT. Highly expressed MARCO TAMs cause GSCs to phenotypically change to a mesenchymal form, boosting invasion and proliferation activities as well as radiation treatment resistance.^90^^,^^91^ Also, it greatly sped up *in vivo* tumor implantation and development.^90^ Moreover, when PTEN is lost, the PI3K pathway is more commonly abnormal in TAM cancers with high levels of MARCO.^90^

#### EPO and EPOR of microglia and oligodendrocytes

EPO also mediates paracrine pathways.^59^ Additionally, the mesenchymal characteristics of GBM are mediated by the EPO secreted by GBM, which can act on the EPOR of microglia and oligodendrocytes in the microenvironment, promote tumor survival, proliferation, and angiogenesis.^57^^,^^92^

### Paracrine pathways of TAMs promoting GBM

#### OPN of TAMs and integrin αVβ3 of GBM

As previously mentioned, LOX mediates OPN secretion.^85^ OPN released by M2 TAMs is a ligand of integrin αVβ3 receptors in GBM cells.^88^ OPN/integrin αVβ3 paracrine pathway attenuated tumor cell death.^53^^,^^88^ Wei et al discovered that the paracrine mode of interaction between a subset of OPN^+^ TAMs and cancer cells promoted GBM mesenchymal transition and that this effect is related to hypoxia.^93^

#### TGF-β1 of TAM and TGF-βR2 of CD133^+^ GBM

Transforming growth factor-β1 (TGF-β1), an immunosuppressor, is primarily secreted by microglia in TAMs.^94^ M2-type TAMs have been shown to have a significant impact on TGF-β1 secretion, and microglia and monocyte-macrophages are found to be the major contributors to early and late secretion.^94^ In the paracrine pathway, TGF-βR2 on CD133^+^ GSC can bind with TGF-β1 released by TAMs, and mediate a number of responses.^94^ Firstly, it promoted GBMs' MMP-9 expression and invasion.^94^^,^^95^ Secondly, TGF-βR2 promoted GBM self-renewal by transactivating TGF-βR1, which then activates ERK, PI3K-Akt, and p38 intracellular signals.^94^ These facts imply that TGF-β1 secreted by TAMs, is crucial for sustaining and developing mesenchymal features.

#### PTN of TAM and PTPRZ1 of GBM

A liver-binding glycoprotein called polytrophin (PTN) is expressed by the CD11b^+^ CD163^+^ M2 TAMs, stimulating the growth and proliferation of cancer cells by binding to the protein PTPRZ1 (protein tyrosine phosphatase, receptor type Z1) on GBM cells.^96^ Studies have shown that PTN-PTPRZ1 signaling controls the phosphorylation of Fyn, activating the AKT pathway for the maintenance of GSCs.^97^ The PTN-PTPRZ1 paracrine pathway induced by M2 TAMs seems to be another manner in which M2 TAMs facilitate GBM mesenchymal transition.^97^

#### OSM of TAMs and OSMR, LIFR, IL-6R of GBM cell or PDGFRA^+^ oligodendrocytes

Oncostatin M (OSM) is a member of the IL-6 family. During routine bodily functions, neutrophils and macrophages release OSM.^98^ OSM is mostly secreted in inflammatory settings, and studies have shown that it is related to the growth of malignancies and has a significant function in PMT.^99^ OSM possesses the most comprehensive downstream signaling pathways of the IL-6 family, including the Jak-STAT3, ERK1/ERK2, PI3K/Akt, NF-B, and Jak-STAT3 pathways.^99^^,^^100^ The most frequently mentioned OSM downstream signaling pathway among them is Jak-STAT3.^101^ In the GBM milieu, microglia in TAMs are primarily responsible for secreting OSM. In line with this, its receptors OSMR and LIFR are expressed in PDGFRA^+^ oligodendrocytes and GBM tumor cells.^101^

OSM-OSMR is a critical pathway for promoting tumor cell malignancy. Hara et al demonstrated that OSM activates STAT3 by binding to OSMR, LIFR, and IL-6R, and subsequently triggers various intracellular pathways.^102^ The normal function of STAT3 is essential for this process. Studies showed that several chemokines, including CCL3/3L1/4/4L2/5/7/8, CSF1, and CXCL2/3/8, co-express with OSM,^101^ indicating that these chemokines might play a role in PMT.^103^ In conclusion, the OSM-OSMR pathway promotes PMT depending on the crosstalk between GBM and TAMs.

### Paracrine pathways between GBM and endothelial cells

Angiogenesis, particularly in the MES subgroup of GBM, results from communication between GBM and nearby vascular endothelial cells. These cells, along with TAMs, have a paracrine network induced by ligand/receptor interactions with GBM in the TME.^104^^,^^105^ As a result, a method for understanding PMT may also be found in the paracrine pathway of endothelial cells and GBM.

### Paracrine pathways of GBM promoting angiogenesis

#### CXCL-8 and CXCR2 of endothelial cells

Shen et al found that CXCL-8 plays a critical regulatory function in angiogenic tissue repair by inducing vascular endothelial cells to secrete cytokines like VEGF and SOD when skin injury happens.^106^ Through binding to CXCR2 on endothelial cells, CXCL-8 secreted by GBM cells promotes GBM angiogenesis and mesenchymal characteristics.^107^ Moreover, up-regulation of Bcl-xL protein in GBM triggers the CXCL-8/CXCR2 axis, which leads to the stimulation of biological processes like cell proliferation, endothelial cell migration, and the formation of vital vascular structures in the central lumen.^43^

#### VEGF and VEGFR of endothelial cells

Vascular endothelial growth factor (VEGF) possesses a proangiogenic impact that has been well documented.^108^ Along with an autocrine cycle, VEGF also mediates paracrine pathways. VEGF released by GBM interacts with VEGFR of vascular endothelial cells to increase angiogenesis and vascular permeability, which facilitates the transformation of the tumor into a mesenchymal phenotype.^36^

#### CXCL-12 and CXCR4/7 of endothelial cells

As previously stated, CXCL12 and its receptor, CXCR4, were directly related to hypoxia.^47^ In addition to driving angiogenesis in GBM through autocrine activation of PMT-related pathways, CXCL12 secreted by GBM also interacts with CXCR4 in vascular endothelial cells through paracrine actions.^109^ After this, vascular endothelial cells raise the transcript of CXCR4, which facilitates the binding of CXCL-12 and CXCR4.^110^ Additionally, the migration of GBM tumors induced by CXCL12 also relies on endothelial cells that express CXCR7 in hypoxic environments.^110^

#### FGF5 and FGFR1（IIIc）of endothelial cells

Fibroblast growth factor-5 (FGF5) is another ligand that plays a role in mesenchymal transition through paracrine signaling. FGF5 released by GBM is a vital ligand of FGFR1 (IIIc) in vascular endothelial cells, and the activation of the FGF5/FGFR1 (IIIc) pathway enhances the angiogenesis in GBM.^37^

#### STC1 and CaSR of endothelial cells

STC1, which was already stated, also interacted with CaSR in endothelial cells.^63^ Sakata et al showed that STC1 activated the VEGF signaling pathways of endothelial cells and increased the levels of eNOs, VEGF, and VEGFR2 in both mRNA and protein.^63^ Paracrine STC1 can act on endothelial cells to promote angiogenesis.^63^

#### Ephrin-B2 and EPHB2 of endothelial cells

As previously mentioned, ephrin-B2 also mediates the paracrine pathway. Paracrine ephrin-B2 in GBM cells is regulated by TNC and binds to EPHB2 receptors in vascular endothelial cells, inducing angiogenesis and regulating the mesenchymal transition of GBM.^68^

### Paracrine pathways of endothelial cells on GBM progression

#### DLL4/jaggd-1/eNOs, IL-6 of endothelial cells and Notch1/2, CXCR1/2 of GBM

Sharma et al linked four ligands, including DLL4, jaggd-1, eNOs, and IL-6, to endothelial cell-mediated angiogenic effects in their research.^111^ The first three improved the GBM stem cell phenotype by influencing Notch signaling by acting on Notch1/2 receptors, promoting neurosphere formation, and inducing tumorigenicity *in vivo*.^111^ Additionally, CXCR1/2 receptors in GBM cells bound to IL-6 secreted by endothelial cells mediate the formation of additional perivascular niches.^111^ These four binding functions promote the development of perivascular niches, which may pave the way for future research on the mesenchymal transition.

### Paracrine pathways between GBM and others

#### DA and transferrin (TF) of PN-GBM and TFR1 of MES-GBM

Numerous researches have shown that metabolic rewiring of specific tumor cells leads to intra-tumoral metabolic symbiosis, which is also known as “mutualism” and “commensalism”.^112^ This biochemical co-existence promotes tumor heterogeneity by allowing metabolites and signaling molecules to circulate between different kinds of tumor cells.^112^ Vo et al have demonstrated that PN GBM cells secrete DA to foster self-development.^113^ Additionally, through a paracrine mechanism, DA also activates TFR1 receptors in tumor cells with the MES phenotype in a different tumor subtype region. TF then mediates iron uptake by MES subtype cells and lowers the risk of ferroptosis by acting on TFR1.^113^

#### Glu and NMDAR/AMPAR of neurons

GBM-produced Glu stimulates Ca^2+^-permeable NMDAR and AMPAR not only in GBM cells but also in nearby cells, including neurons.^114^ It was demonstrated that this paracrine pathway of Glu acted on nearby neurons' NMDAR/AMPAR receptors, promoted excitotoxic neuronal death, made room for movement, and significantly boosted tumor growth, migration, and tumorigenic potential, eventually promoting mesenchymal phenotype.^114^

#### Kinins of MSCs and kinins-B1/B2 of GBM

The kallikrein-kinin system is an endogenous metabolic pathway that results in the release of kinins, regulating a number of physiological functions.^115^ Pillat et al demonstrated the communication between mesenchymal stem cells (MSCs) and GBM cells is facilitated by kinin ligands secreted by MSCs and the GBM kinin B1 and B2 receptors.^116^ These signals play a role in the migration of GBM cells and angiogenesis.^116^ B1 receptors in particular have been explicitly proven to be crucial for GBM mesenchymal phenotype.^116^

#### C5a of MSCs and C5aR1 of GBM

Complement C5a, which MSCs secrete, is both a chemokine for GBM and a molecule with mesenchymal characteristics.^117^ According to the study of Lim et al, C5a binding to GBM C5aR1 induces the elevation of ZEB1, which is a crucial regulator of the mesenchymal process.^117^ The p38 MAPK pathways are then activated by ZEB1 to promote invasiveness and shift to mesenchymal phenotype.^117^

#### IL-6/IL-11 of CAFs and IL-6R/IL-11R of GBM

CAFs (cancer-associated fibroblasts) are responsible for GBM mesenchymal characteristics.^118^ Studies have demonstrated that CAFs cause the release of interleukins like IL-6 and IL-11 interact with IL-6R and IL-11 on GBM cells, activating the intracellular JAK/STAT3 pathway or the CXCL12/CXCR4 signaling pathway and increasing the activity of cancer cells in terms of invasion, migration, and metastasis.^118^^,^^119^

#### CXCL-12 of CAFs and CXCR4 of GBM

CAFs in the tumor microenvironment secrete more CXCL12 as a result of increased hypoxia, which causes GBM to express more CXCR4 (as described previously). Additionally, the CXCL12/CXCR4 axis can recruit arterial endothelial cells directly, aiding in angiogenesis and mediating the mesenchymal transition.^47^

#### HA of ECM and CD44, RHAMM of GBM

Hyaluronic acid (HA), which is prevalent in invasive cancer cells, makes up a significant portion of the brain ECM.^29^ According to So et al, HA interacted with the GBM CD44-mediated hyaluronic acid mediates movement receptor (RHAMM) to promote the growth, migration, and invasion of GBM.^29^ Moreover, HA plays a role in MMP secretion, indicating a connection to mesenchymal transition.^29^^,^^120^

## Therapies targeting autocrine and paracrine pathways inducing proneural–mesenchymal transition

The increasingly interaction complex mechanism between GBM and the microenvironment has led to the development of receptor and ligand-related drugs that target the microenvironment.^121^ These therapies inhibit PMT-related targets, such as EGFR, TGF-β, VEGF, PDGFR, and FGFR (Table 3).^121^ However, the blood–brain barrier significantly hinders drug delivery and effectiveness for GBM in the brain.^121^ Clinical research on GBM has focused on studying cases with recurrence, refractoriness, and drug resistance, which may have acquired a mesenchymal phenotype. Here are some recent clinical study findings on targeted therapeutic medicines from autocrine and paracrine perspectives.

**Table 3:** Therapies targeting autocrine and paracrine pathways inducing proneural–mesenchymal transition.

Target	Agent	Clinical trial (reference)
EGFR	Afatinib	Phase II in unselected recurrent GBM: manageable safety profile but limited single-agent activity (ClinicalTrials.gov NCT01743950)^126^
	Depatuxizumab mafodotin	Phase III in newly-diagnosed GBM: no OS benefit and no new important safety risks (ClinicalTrials.gov NCT02573324)^127^
		Phase II in recurrent EGFR-amplified GBM: had no impact on HRQoL and NDFS (ClinicalTrial.gov NCT02343406)^128^
	Pulse high-dose lapatinib	Phase II in newly-diagnosed GBM: tolerable and safe regimen, but higher rates of lymphopenia should be noted (ClinicalTrial.gov NCT01591577)^129^
	Anti-EGFR-immunoliposomes	Phase II in EGFR-amplified GBM: showed active but warrant further clinical evaluation (ClinicalTrial.gov NCT03603379)^130^
	Rindopepimut	Phase III in newly diagnosed, EGFRvIII-expressing GBM: did not increase survival (ClinicalTrials.gov NCT01480479)^132^
TGF-β	Galunisertib	Phase II of with Galunisertib with temozolomide-based radiochemotherapy (TMZ/RTX) in newly diagnosed malignant GBM: no differences in efficacy, safety or pharmacokinetic variables were observed between the two treatment arms (ClinicalTrials.gov NCT01220271)^133^
	Trabedersen	Phase II in high-grade glioma: 10 μM is the optimal dose (ClinicalTrials.gov NCT00431561)^134^
VEGF	Bevacizumab	Phase II of bevacizumab with either irinotecan in recurrent GBM: showed therapeutic benefit (ClinicalTrials.gov NCT00433381)^135^
		Phase II of bevacizumab + dose-dense temozolomide in recurrent GBM: showed confirming activity (ClinicalTrials.gov NCT00433381)^135^
		Phase II of bevacizumab + vorinosta in recurrent GBM: did not yield improvement in PFS, OS or clinical benefit (ClinicalTrials.gov NCT01266031)^137^
		Phase II of bevacizumab + trebananib in recurrent GBM: showed minimal activity (ClinicalTrials.gov NCT01609790)^138^
		Phase III of bevacizumab + lomustine in progressive GBM: did not confer a survival advantage over treatment with lomustine alone (ClinicalTrials.gov NCT01290939)^139^
	Ponatinib	Phase II in bevacizumab-resistant GBM: limited efficacy (ClinicalTrials.gov NCT02478164)^136^
	Aflibercept	Phase II in recurrent malignant GBM: moderate toxicity and minimal evidence of single-agent activity (ClinicalTrials.gov NCT00369590)^140^
FGFR	Nintedanib	Phase II in recurrent GBM regardless of prior bevacizumab therapy: no active (ClinicalTrials.gov NCT01380782)^141^
	Dovitinib	Phase II in recurrent GBM: was not efficacious in prolonging the PFS (ClinicalTrials.gov NCT01753713)^142^
PDGFR	Nintedanib	Phase II in recurrent GBM: well tolerated and clinically non-relevant antitumor activity (ClinicalTrials.gov NCT01380782)^141^ Phase II in recurrent GBM: not active (ClinicalTrial.gov NCT01251484)^143^
	Dasatinib	Phase II in recurrent GBM: ineffective (ClinicalTrials.gov NCT00423735)^144^

### Therapies targeting autocrine pathways

#### EGFR

A popular molecule for targeted treatment is the epidermal growth factor receptor (EGFR), which is connected to PMT.^122^ The EGFRvIII mutant indicates a poor prognosis for GBM.^123^ Research has focused on immunotherapy and targeted medications, such as Afatinib, which demonstrated limited single-agent activity in unselected individuals with recurrent GBM.^124^ Depatuxizumab mafodotin (depatux-m) did not provide survival benefits over placebo in phase III clinical study.^125^ A 2021 trial using depatux-m to treat EFGR-amplified recurrent GBM had no success.^126^ Pulse high-dose lapatinib is a safe regimen for newly-diagnosed GBM, but it may cause lymphopenia.^127^ Immunotherapy, such as EGFRvIII-targeted peptide vaccines or anti-EGFR immunoliposomes, has shown progress in some patients but not all.^128^^,^^129^ Rindopepimut, an anti-EGFRvIII vaccine, did not extend survival time in patients with freshly diagnosed glioblastoma.^130^ Targeted treatment for EGFR requires more study, particularly in relapsed or refractory patients.

### Therapies targeting paracrine pathways

#### TGF-β

The paracrine-related molecule TGF-β, which is closely linked to mesenchymal transformation, was previously noted.^94^ Studies targeting TGF-β are also being done, but they are still tiny and ineffective.^131^^,^^132^ One explanation is that there is a greater interplay between this molecule's release and acceptance, and as a result, the therapeutic therapy impact is not what was anticipated.

### Therapies targeting autocrine and paracrine pathways

#### VEGF

VEGF is a key molecule in the angiogenesis of MES-GBM, and previous clinical studies have targeted its paracrine effect with the monoclonal antibody bevacizumab.^121^ Recent research focuses on combining bevacizumab with other treatments, such as irinotecan and temozolomide.^133^ However, other VEGF-targeting combinations like ponatinib, vorinostat, and trebananib appear ineffective, and trebananib may even have harmful interactions with bevacizumab.^134^, ^135^, ^136^ Another research that attempted to establish whether the addition of bevacizumab would result in patients with initial progression of malignant gliomas having longer overall survival than lomustine alone came up empty-handed.^137^ Patients with recurrent glioblastoma can use aflibercept (VEGF trap), a recombinant fusion protein that eliminates VEGF and placental growth factors.^138^ However, a study found that aflibercept monotherapy has moderate toxicity and little indication of single-agent activity in unselected patients with recurrent malignant glioma.^138^ In conclusion, a more thorough study is still needed on VEGF-targeted therapy for GBM.

#### FGFR

The available targeted pharmacological therapy for FGFR is currently limited, even though FGFR is also a common effector receptor of autocrine and paracrine.^37^ Nintedanib showed no effect on relapsed high-grade gliomas, regardless of prior bevacizumab therapy, according to Norden et al's investigation of these tumors.^139^ Dovitinib was found to not affect PFS in patients with relapsed GBM despite early anti-angiogenic therapy (including bevacizumab), according to another trial of relapsed gliomas at the same target.^140^ In actuality, neither of these two medications particularly targets FGFR. Poor clinical benefit could have several causes, including low specificity.

#### PDGFR

The PDGFR autocrine pathway in GBM is the subject of some clinical research as well. Targeting VEGFR1-3, FGFR1-3, and PDGFR-a/b, nintedanib (BIBF 1120) is a small, orally accessible, triple angiokinase inhibitor that is in phase III development.^139^ However, in patients with recurrent GBM who had not responded to 1/2 lines of prior therapy, single-agent nintedanib (200 mg bid) showed limited but clinically insignificant antitumor activity.^139^ Additionally, Muhic et al attested to nintedanib's ineffectiveness.^141^ Another medication that targets PDGFR is dasatinib. However, despite efforts to broaden the community and raise the dose, Dasatinib was unable to show efficacy as a monotherapy for recurrent GBM, according to research by Lassman et al.^142^

All in all, most clinical studies indicate that the prospects for the use of targeted drugs in the therapy of GBM are dim. The mechanism of immunotherapy resistance may involve the fluctuating and uncontrollable spliceosome and an immunosuppressive microenvironment. Additionally, the multithreading of receptor ligands may also contribute to drug resistance.^6^ If there is a promising future for molecularly targeted drugs with other PMT-related receptor ligands in the microenvironment, more clinical practice and study are required.

## Conclusion

GBM treatment resistance remains a challenge, and new strategies are needed to confront its heterogeneity and replace traditional cytotoxic chemotherapy. Technologies such as scRNA-seq and spatial transcriptome sequencing have increased our understanding of tumor heterogeneity. This review shows that GBM achieves PMT through complicated autocrine and paracrine systems, with multiple signals and various types of cells that mutually complement each other. Inhibiting a single target or pathway might not be sufficient.

GBM medication resistance induced by PMT is influenced by various extrinsic and intrinsic factors, including tumor heterogeneity, hypermutation, altered metabolomics, and oncologically activated alternative splicing pathways. In addition, immunotherapy often fails in GBM due to hypoxia and an immune-suppressive tumor microenvironment. Efforts are currently focused not only on reducing immunotolerance but also on preventing tumor cell escape mechanisms from treatment, which are caused by inter- and intra-tumoral heterogeneity.

To overcome the treatment resistance resulting from intricate autocrine and paracrine systems, two potential directions are worth studying: one is to explore novel effective therapeutic regimens, and the other is to combine multiple therapies. As we summarized, TAMs play a crucial role in PMT through paracrine manner, immunotherapy especially for TAMs including engineered immune cells such as chimeric antigen receptor-macrophage (CAR-M) might be a promising approach.^143^ Preclinical studies of combination therapies were also performed to confront the PMT. What's more, combination strategies are attracting accumulating attention. For example, dual targeting of polyunsaturated fatty acid synthesis and EGFR signaling has shown a combinatorial cytotoxic effect on GSCs.^144^ Our former study also demonstrated that the combination of targeting spliceosome and NF-κB therapy significantly inhibited PMT.^145^ Although the security of these combination therapies still needed to be confirmed, a combination strategy might be prospective to overcome the mechanisms of mutual complement in the autocrine and paracrine systems.

The proposed receptor–ligand interaction pathways in this review highlight the complexity of the GBM microenvironment, involving various molecules such as neurotransmitters, chemokines, hormones, growth factors, and secreted glycoproteins. We classified these ligands and described their associations with PMT. In addition to receptors and ligands, cell-to-cell connections also include direct contact, gap junctions, intercellular nanotube tunnels, and exosomal vesicles, which require further in-depth study.

In conclusion, this study reviews the molecular pathways of PMT mediated by receptor and ligand binding in the tumor microenvironment, and it is hoped to provide help and support for future work in related fields.

## Author contributions

Y.L.3 and S.Q. contributed to the design and supervision. Y.L.1, X.L., and Y.L.2 contributed to the writing and editing of the manuscript. P.O., H.W., X.Z., and G.H. contributed to the data collection.

## Conflict of interests

The authors have declared that no competing interest exists.

## Funding

This study was supported by the 10.13039/501100001809National Natural Science Foundation of China (No. 82203368), Science and Technology Projects in Guangzhou, Guangdong, China (No. 202201011008), and College Students' Innovative Entrepreneurial Training Plan Program, China (No. 202112121201).
